# Supplementary material for: Potential Application of Egg White Peptides for Antioxidant Properties: Perspectives from Batch Stability and Network Pharmacology
Source: Foods. 2024 Oct 2;13(19):3148. doi: 10.3390/foods13193148 (PMC11475914; doi:10.3390/foods13193148)
Supplement: Supplementary file 1 [file foods-13-03148-s001.zip › foods-3173589-supplementary.pdf]

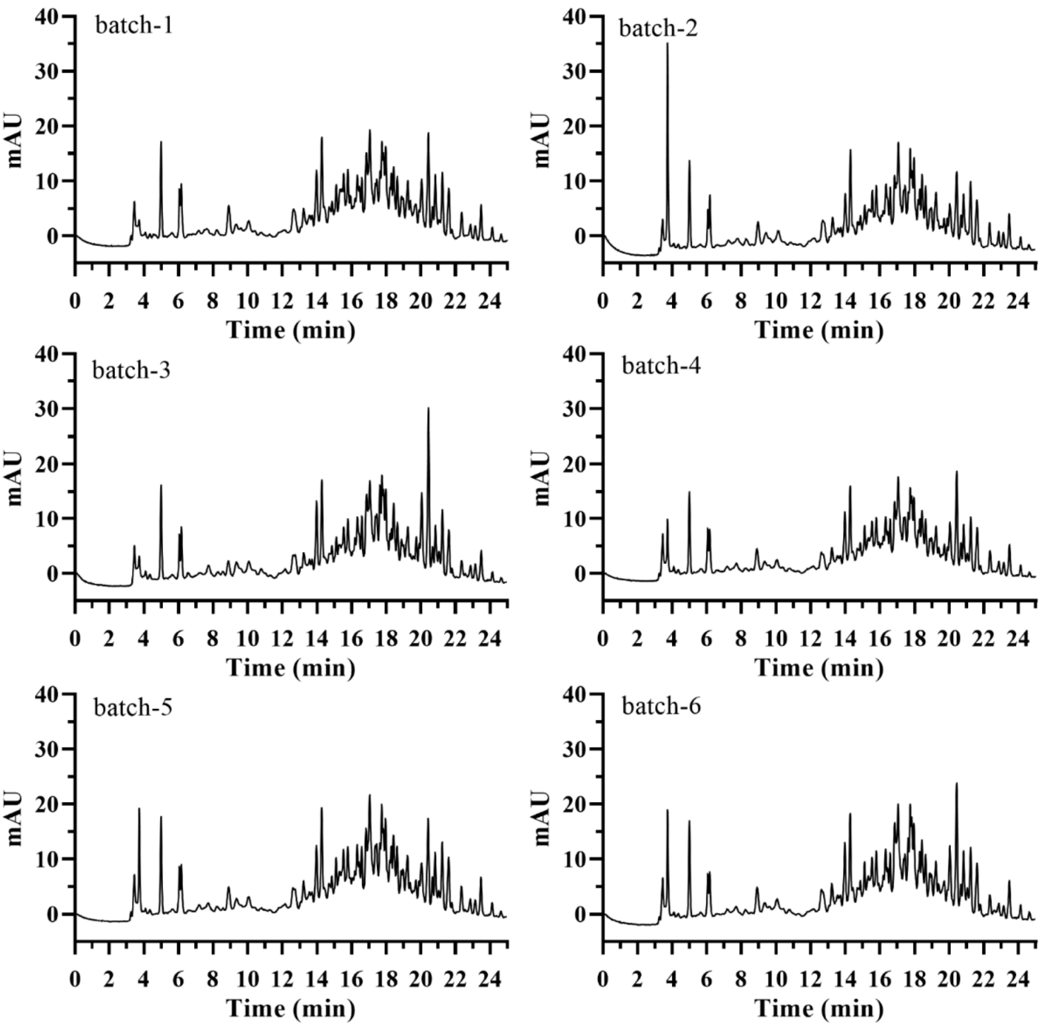

Figure S1 HPLC chromatograms of six batches of egg white peptides (EWPs). “1-6” means different batches.

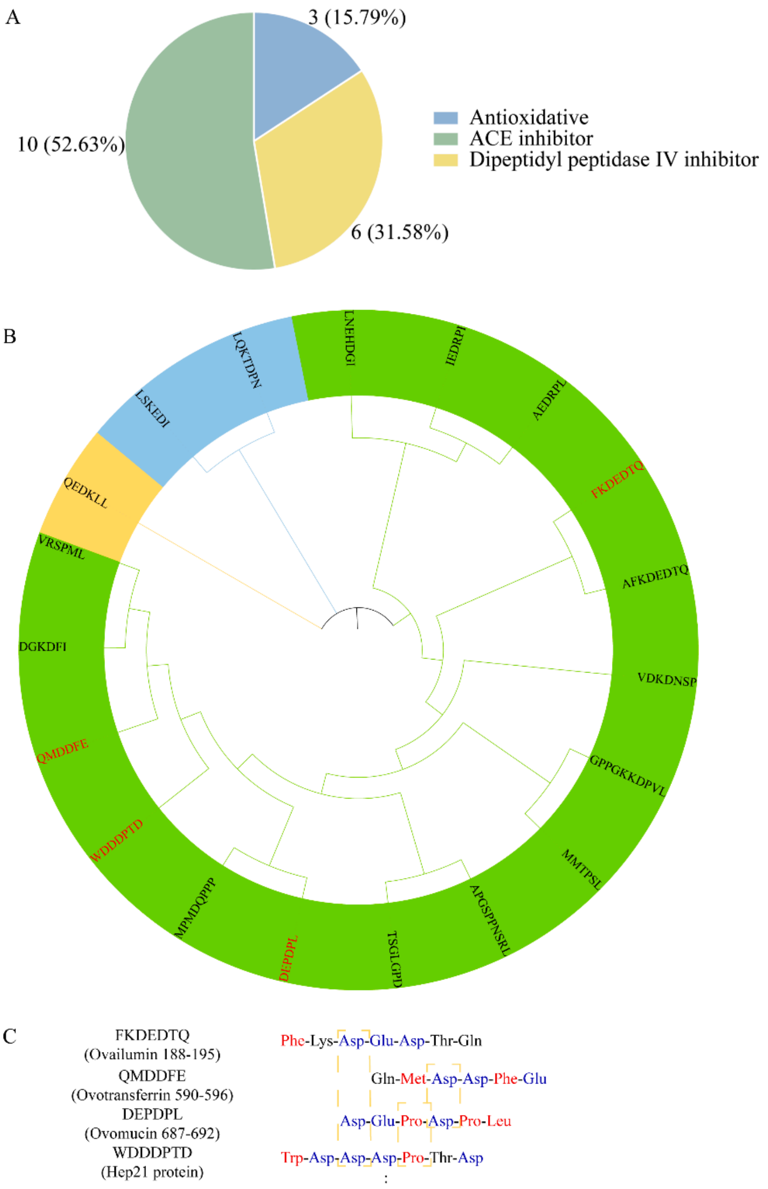

Figure S2 Bioinformatic analysis of composing peptides in egg white peptides (EWPs). (A) The proportion of bioactive peptides in EWPs. (B) Non-redundant peptide sequences were subjected to multiple sequence alignment analysis using the Clustal Omega program and then to the construction of a phylogenetic tree using the iTOL online tool. Different color represents different clade. (C) Comparison of amino acid residues of EWPs. The red letters are hydrophobic amino acids, the blue letters are acidic amino acids, and the same amino acid sequence was marked with the yellow dotted line, ":" means that the letters in this column are either the same or similar.

Table S1 Free amino acid content in egg white peptides.

Amino acid \ Batch		1	2	3	4	5	6
**Essential amino acid (EAA)/μg/mL**	Trp	1.5286	1.6587	2.4461	1.9998	1.1441	2.6285
	Ile	4.1217	2.3535	2.6963	1.9491	2.0007	4.4207
	Phe	4.7907	4.1263	6.6177	4.4659	4.3617	5.5220
	Leu	5.8481	4.1223	5.3225	3.8000	3.9755	4.7997
	Val	4.9834	4.2139	4.2327	4.2635	4.3537	6.5692
	Thr	5.6779	5.5300	5.9810	5.2666	5.4607	5.9014
	Met	5.2364	15.1017	7.0094	4.7245	4.8859	5.8044
	Lys	11.5500	16.4791	12.0662	10.7913	12.6470	12.3427
**Non-essential amino acid (NEAA)/μg/mL**	His	1.9304	1.5524	1.8459	1.5336	1.7646	1.7810
	Gly	1.3848	1.5551	0.7750	1.1168	1.0686	1.4099
	Ser	1.7081	1.4693	1.8998	1.4887	1.8554	1.6884
	Asp	0.9487	1.3671	2.0935	1.4367	1.6533	1.5232
	Asn	3.8237	2.0713	2.4435	2.9923	3.1997	3.3225
	Arg	7.7814	7.6258	7.6258	6.0776	4.7337	7.2758
	Ala	12.8317	10.5017	11.4499	7.8430	10.5179	3.3297
	Gln	14.4507	6.5881	13.9469	11.8790	13.6702	14.5361
	Glu	18.0761	16.2021	16.1302	14.5335	15.4772	15.1531
	Pro	17.8575	14.3574	16.1302	15.5975	15.9336	16.6703
	Tyr	16.1884	21.7495	21.5588	17.9962	21.7116	20.9309
	Cys	21.6463	9.7490	8.6697	17.3835	21.0138	20.1028
**Total amino acid (TAA)/μg/mL**		162.3646	148.3743	150.9411	137.1391	151.4289	155.7123

"1-6" means different batches.

Table S2 *In vitro* antioxidant activity of egg white peptides (EWPs).

Batch	ABTS						FRAP	ORAC
	Antioxidant fitting equation	R^2	IC_{50} (mg/mL)	Trolox standard curve equation	R^2	Trolox equivalent (mg/mL TE / mg/mL)	Fe equivalent （mg/mL Fe / mg/mL）	Trolox equivalent (mg/mL TE / mg/mL)
1	y=32.031ln(x)+91.016	0.9927	0.2899±0.0245ab	y=0.9888x-0.172	0.9997	0.0924±0.0090a	0.9862±0.048ab	0.2255±0.0300a
2			0.2950±0.0234ab			0.0903±0.0004a	0.9380±0.0119b	0.2031±0.0232a
3			0.2945±0.0320ab			0.0908±0.0010a	1.0143±0.0165ab	0.2046±0.0231a
4			0.3075±0.0235a			0.0906±0.0004a	1.0207±0.0341a	0.1734±0.0199ab
5			0.2611±0.0161b			0.0925±0.0009a	0.9816±0.0287ab	0.1780±0.0151ab
6			0.3339±0.0200a			0.0870±0.0014b	0.9443±0.0322ab	0.1336±0.0151b

Tips: significant differences at the level of $p < 0.05$.

Table S3 Information of the egg white peptides (EWPs) sequence, "1-6" means different batches.

Batch	No.	Sequence	Length	Proteins	m/z	Mass	Score
1	1	QMDDFE	6	P02789	784.2818	783.2745	140.0100
	2	WDDDPTD	7	Q8AV77	863.3054	862.2981	106.7600
2	1	FKDEDTQ	7	P01012	441.6956	881.3767	134.8100
	2	WDDDPTD	7	Q8AV77	863.3054	862.2981	132.9400
	3	QMDDFE	6	P02789	784.2818	783.2745	124.3200
	4	DEPDPL	6	Q98UI9	685.3039	684.2966	95.3780
	5	LSKEDI	6	F1NWP3	352.6949	703.3752	71.0670
	6	QEDKLL	6	Q98UI9	745.4091	744.4018	57.8110
	7	DGKDFI	6	Q6JGV1	347.6740	693.3334	57.1020
	8	IEDRPI	6	Q766V2	371.7083	741.4021	54.8950
3	1	FKDEDTQ	7	P01012	441.6956	881.3767	93.4290
	2	VRSPML	6	F1NMJ6	702.3967	701.3894	92.2790
	3	GPPGKKDPVL	10	P02789	504.2978	1006.581	92.2390
	4	MMTPSL	6	P21642	348.1588	694.303	52.5890
4	1	DEPDPL	6	Q98UI9	685.3039	684.2966	117.5400
	2	QMDDFE	6	P02789	784.2818	783.2745	101.3300
	3	LNEHDGI	7	Q98UI9	399.1930	796.3715	68.7870
	4	QEDKLL	6	Q98UI9	745.4091	744.4018	62.9780
5	1	DEPDPL	6	Q98UI9	685.3039	684.2966	103.4100
	2	APGSPPNSRL	10	P02789	498.2671	994.5196	102.0600
	3	LNEHDGI	7	Q98UI9	399.1930	796.3715	79.4510
	4	MMTPSL	6	P21642	711.3052	710.2979	51.9030
6	1	FKDEDTQ	7	P01012	441.6956	881.3767	148.0400
	2	WDDDPTD	7	Q8AV77	863.3054	862.2981	134.1200
	3	QMDDFE	6	P02789	784.2818	783.2745	124.3200
	4	VRSPML	6	F1NMJ6	702.3967	701.3894	92.0750
	5	AEDRPL	6	B6V1G0	350.6849	699.3552	89.6730
	6	AFKDEDTQ	8	P01012	477.2142	952.4138	88.8190
	7	DEPDPL	6	Q98UI9	685.3039	684.2966	88.6270
	8	TSGLGPD	7	P20740	646.3043	645.2970	79.9390
	9	MPMDQPPP	8	A0A1L1RRM6	456.7013	911.3881	71.3420
	10	LQKTDPN	7	Q9DER4	815.4258	814.4185	60.2940
	11	VDKDNSP	7	P20740	387.6851	773.3556	55.4410

Table S4 Topological analysis of the protein-protein interaction (PPI) network

Name	Betweenness Centrality	Closeness Centrality	Clustering Coefficient	Degree
AKT1	0.622	0.605	0.103	23
GSTP1	0.113	0.474	0.489	10
GSTM1	0.128	0.474	0.583	9
SULT1A1	0.078	0.469	0.472	9
GSTA1	0.009	0.362	0.714	8
INSR	0.043	0.438	0.286	7
LCK	0.008	0.414	0.524	7
RAC1	0.007	0.407	0.429	7
BTK	0.044	0.407	0.333	7
GSTZ1	0.020	0.351	0.714	7
GSTM2	0.001	0.357	0.905	7
GSTT2B	0.001	0.357	0.905	7
EIF4E	0.045	0.430	0.333	6
ATIC	0.062	0.400	0.200	6
DUT	0.130	0.354	0.067	6
GSTA3	0.000	0.348	1.000	6
RASA1	0.002	0.400	0.700	5
UCK2	0.131	0.460	0.500	5
CASP1	0.024	0.404	0.400	5
PAPSS1	0.032	0.374	0.300	5
NT5M	0.057	0.362	0.400	5
SULT2A1	0.012	0.365	0.400	5
MAPK12	0.001	0.393	0.667	4
GSK3B	0.001	0.400	0.667	4

IMPDH1	0.028	0.434	0.667	4
GLO1	0.037	0.383	0.000	4
SULT2B1	0.002	0.351	0.667	4
SELE	0.001	0.390	0.667	3
ANG	0.041	0.400	0.000	3
RND3	0.000	0.387	1.000	3
ALAD	0.052	0.371	0.000	3
WARS	0.056	0.305	0.000	3
RNASE2	0.003	0.303	0.333	3
LYZ	0.001	0.305	0.667	3
PMS2	0.000	0.383	1.000	2
MME	0.000	0.390	1.000	2
MMP12	0.000	0.383	1.000	2
FGG	0.004	0.319	0.000	2
DDX39B	0.014	0.338	0.000	2
HAGH	0.001	0.293	0.000	2
RAP2A	0.000	0.380	0.000	1
FDPS	0.000	0.380	0.000	1
PFKFB1	0.000	0.380	0.000	1
RFK	0.000	0.263	0.000	1
IVD	0.000	0.263	0.000	1
NMNAT3	0.000	0.267	0.000	1
TAP1	0.000	0.235	0.000	1
